# Spiritual and Religious Connections to Reducing Risk of Comorbidity Among Middle Aged Women in Correctional Custody

**DOI:** 10.1093/geroni/igaf122.3131

**Published:** 2025-12-31

**Authors:** Serena Gray, Alex Bishop

**Affiliations:** Oklahoma State University, Stillwater, Oklahoma, United States; Oklahoma State University, Stillwater, Oklahoma, United States

## Abstract

Data for this study originated from a sample of N = 220 middle-aged women, aged 40 and older (M = 49.05, SD = 7.23), under state correctional custody in Oklahoma. Logistic regression analyses were computer to examine the relationship of socio-demographic characteristics, adverse childhood experiences, mental health, and religious and spiritual connections to comorbid health impairment. Results indicate that age, (OR = 1.16, CI: 1.03-1.29), attachment to God (OR = 1.11, CI: 1.03-1.18), and religiosity (OR = 1.28, CI: 1.09-1.50) are associated with increased odds of comorbid health impairment; whereas forgiveness was associated with reduced odds (OR = .96, CI: .92-1.00). Middle-aged women who continue to age while in correctional custody appear to be at a greater risk for experiencing comorbid health problems. Yet, a greater disposition to engage in forgiveness may help off-set this risk. Findings further suggest that a desire to strengthen a relationship and make things right with God, as well as become more actively involved in religious activities may signifying behaviors aimed at resolving increased occurrence of comorbid health impairments. Study results have implications relative to how correctional case managers, forensic social workers, and prison chaplains can better identify and meet the religious, spiritual, and health needs of middle-aged women under correctional custody.

